# Partial omentectomy maybe practicable for T3 or shallower gastric cancer patients

**DOI:** 10.1002/cam4.4980

**Published:** 2022-07-20

**Authors:** Qiaowei Lin, Qianming Bai, Qiuyi Huang, Yakai Huang, Jianpeng Gao, Yu Zhang

**Affiliations:** ^1^ Department of Gastric Surgery, Shanghai Cancer Center Fudan University Shanghai China; ^2^ Department of Oncology, Shanghai Medical College Fudan University Shanghai China; ^3^ Department of Pathology, Shanghai Cancer Center Fudan University Shanghai China

**Keywords:** gastric cancer, omentectomy, prognosis, TNM stage, tumor deposits

## Abstract

**Background:**

Total omentectomy is often performed with gastrectomy as radical surgery for gastric cancer (GC) patients. However, it remains controversial whether GC patients can benefit from omentectomy. The aim of this study was to analyze the incidence and clinical significance of tumor deposits (TDs) in different anatomical subregions of perigastric omentum in GC patients undergoing gastrectomy with total omentectomy.

**Methods:**

From October 2011 to December 2013, 1253 patients who underwent gastrectomy with total omentectomy for GC were retrospective reviewed. The TDs in different anatomical subregions of perigastric omentum were examined.

**Results:**

Of 1253 patients, TDs positivity was 11.2%. Tumor deposits in the omentum of greater curvature and in the omentum of lesser curvature were associated with lymphovascular invasion, perineural invasion, advanced tumor node metastasis stages, and unfavorable survival. Besides, TDs in the proximal omentum of greater curvature and in the omentum of lesser curvature correlated with older patients and larger tumors. Kaplan–Meier curves showed that patients with TDs had worser overall survival (OS) than those without, regardless of TD positions. Patients with TDs in the omentum of greater curvature had the worst prognosis, followed by patients with TDs in the omentum of lesser curvature and patients with no TDs. Tumor deposits in the proximal omentum of greater curvature was an independent prognostic factor for OS. Moreover, only patients classified as pT4 had TDs in the distal omentum of greater curvature.

**Conclusions:**

Patients with TDs in the omentum of greater curvature had the worst prognosis, followed by patients with TDs in the omentum of lesser curvature and patients with no TDs. In addition, partial omentectomy might be practicable for gastric cancer patients classified as T3 or shallower tumors.

## Graphical abstract

Only patients classified as pT4 in gastric cancer had tumor deposits in the distal omentum of greater curvature, indicating that partial omentectomy might be practicable for gastric cancer patients classified as T3 or shallower tumors.

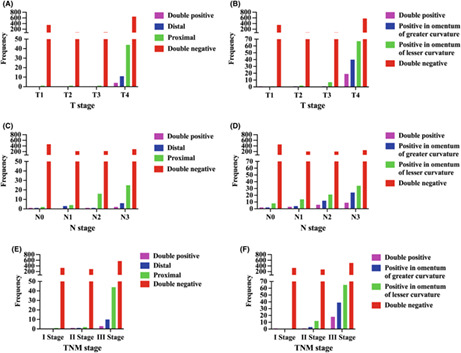

## 1 INTRODUCTION

Gastric cancer (GC), which is the fifth most common malignant tumor and the fourth leading cause of cancer‐related deaths worldwide, is a global challenge to human health.^1^ It is endemic mainly in East Asia and South America and the incidence is increasing in developing countries.^2^ Although diagnostic and therapeutic techniques have developed quickly in recent years, the prognosis of GC remains poor. The 5‐year survival rate of stage IV GC patients is still lower than 15%.^3^ Radical dissection, usually accompanied by chemotherapy or radiation, remains the mainstay therapy for advanced resectable GC and has to some extent improved the survival of GC patients.^4^, ^5^, ^6^, ^7^

Tumor deposits (TDs), first proposed by Gabriel in 1935,^8^ are defined as satellite nodules in the adipose tissue around the primary tumor without residual lymph nodes histologically, which can be characterized by discontinuous spread, venous infiltration with extravascular spread, or complete replacement of lymph nodes. Tumor deposits have been described in many cancers, for example, GC, ovarian cancer, cholangiocarcinoma, pancreatic cancer, colorectal cancer, etc.^9^ Tumor deposits are significant prognostic factors indicating a particularly aggressive biological behavior in several types of cancers such as head and neck,^10^ esophageal,^11^ colorectal,^12^, ^13^ bladder,^14^ and endometrial^15^ cancers. Tumor deposits are incorporated into American Joint Committee on Cancer (AJCC) grading system of colorectal cancer after revealing its prognostic and predictive role.^16^, ^17^ However, the exact role of TDs in GC remains unclear yet.

The greater omentum hangs down from the greater curvature of the stomach and is a double layer of peritoneum separated by a small amount of connective tissue.^18^ The greater omentum plays an important role in peritoneal defense by removing bacteria from the peritoneal cavity.^18^, ^19^, ^20^ In addition, by interposing between the abdominal viscera and the abdominal wall and producing fibrinolytic factors, the greater omentum can reduce postoperative intestinal adhesions.^20^, ^21^, ^22^, ^23^ Milky spots, located mainly in the greater omentum, are composed of numerous macrophages and lymphocyte aggregations.^24^ They are believed to be responsible for the implantation of free cancer cells and the establishment of peritoneal metastasis.^25^ Nowadays, it is thought that complete omentectomy can ensure the elimination of micrometastasis for advanced GC.^18^, ^26^, ^27^, ^28^, ^29^ However, the survival benefit of complete omentectomy remains controversial. The aim of this study is to elucidate the incidence and clinical significance of TDs in different anatomical subregions of perigastric omentum in patient undergoing gastrectomy with total omentectomy for GC.

## 2 MATERIALS AND METHODS

### 2.1 Patients

We retrospectively reviewed and analyzed GC patients who underwent primary gastric surgery at Shanghai Cancer Center, Fudan University from October 2011 to December 2013 (*n* = 1253). The inclusion criteria were as follows (1): primary surgery (gastrectomy with complete omentectomy), (2) pathological diagnosis of adenocarcinoma, (3) no secondary tumor, (4) no distant metastasis, and (5) complete clinicopathologic and follow‐up data. Every patient signed informed written consent. Ethical approval was obtained from the Research Ethics Committee of Shanghai Cancer Center, Fudan University.

### 2.2 Pathological examinations

**FIGURE 1:**
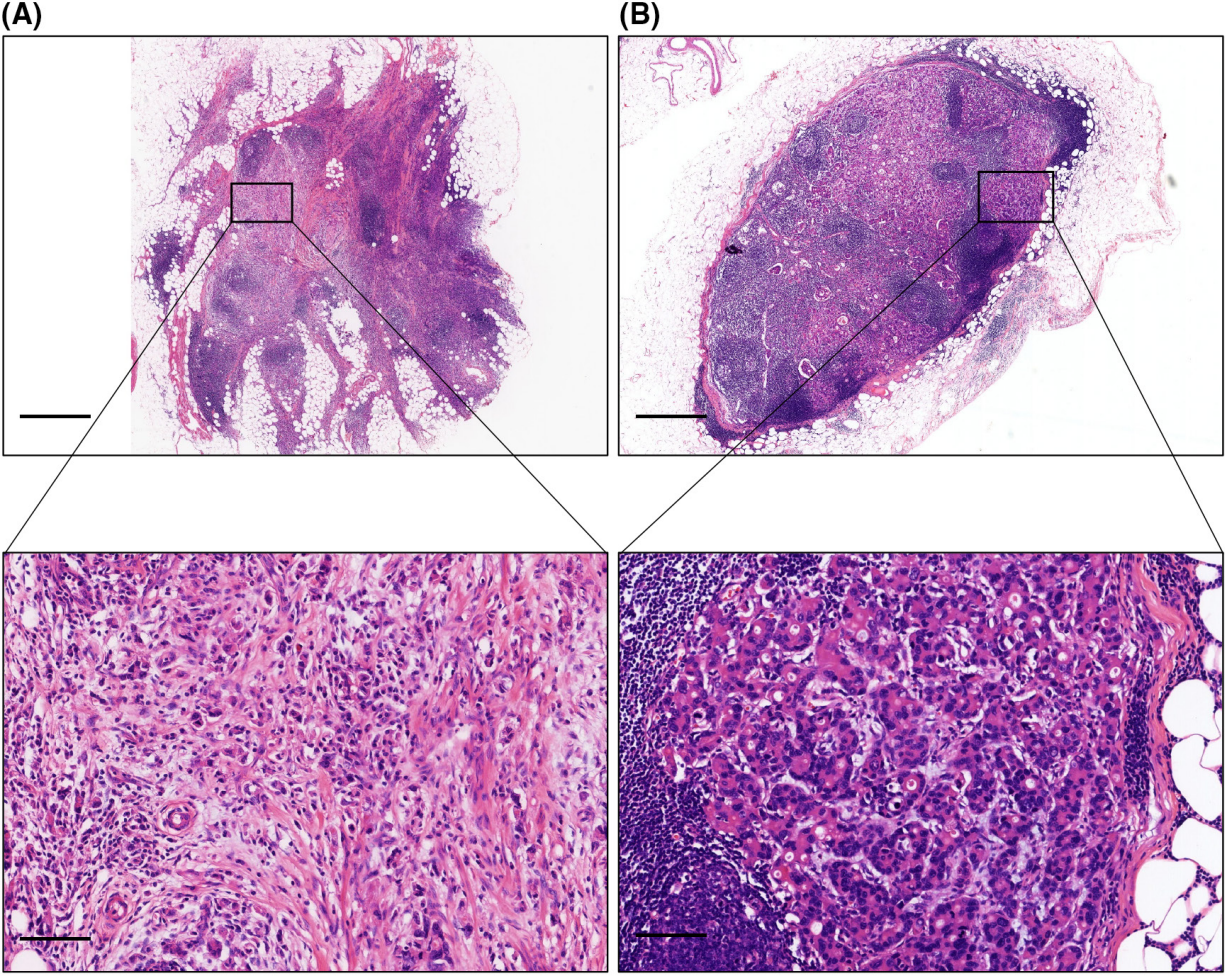
Representative H&E images of GC patient. (A) Representative H&E images of TDs in a GC patient (scale bar: 200 μm [upper], 50 μm [lower]). (B) Representative H&E images of metastatic lymph node in a GC patient (scale bar: 200 μm [upper], 50 μm [lower]). H&E, hematoxylin and eosin; GC, gastric cancer; TD, tumor deposit.

The diagnosis was confirmed by a pathologist who specialized in GC in our center. Pathological features were recorded as follows: tumor location, tumor size, differentiation, lymphovascular invasion, perineural invasion, tumor node metastasis (TNM) stage, surgical margin, and TDs. Based on previous literature, the greater omentum was divided into proximal omentum and distal omentum along gastroepiploic vessels.^18^, ^30^, ^31^ Tumor deposits were classified into three categories based on their anatomical locations, (1) TDs in the omentum of lesser curvature: TDs located in the omentum of lesser curvature of stomach; (2) TDs in the proximal omentum of greater curvature: TDs located in the proximal omentum of greater curvature of stomach; and (3) TDs in the distal omentum of greater curvature: TDs located in the distal omentum of the greater curvature of stomach. In addition, the differentiation of tumor cells referred to the degree of similarity of tumor cells to normal cells. High differentiation indicated that tumor cells were closer to normal cells. Low differentiation meant that tumor cells differentiated far away from normal cells. Medium differentiation indicated that the degree of the differentiation was between high differentiation and low differentiation. The pathologist examined pathological sections of each patient twice to differentiate TDs (Figure 1A) from lymph node metastasis (Figure 1B).

### 2.3 Follow‐up data

Follow‐up data were recorded with the help of electronic medical records of our center. All patients were followed up to either December 2019 or the date of death. Overall survival (OS), which was defined as the time from the date of last follow‐up time or that of death, was the endpoint of this study.

### 2.4 Statistical analysis

IBM SPSS Statistics 23 (SPSS, Inc.) and GraphPad Prism 7.0 (GraphPad Software) software were used to analyze clinical data. Univariate and multivariate analyses were performed using Cox proportional hazards regression to study independent prognostic factors in our cohort. Kaplan–Meier curves were performed to identify differences in OS in various patients who underwent curative surgery. A *p* value <0.05 was considered statistically significant.

## 3 RESULTS

### 3.1 Patient characteristics

Comprehensive clinical characteristics of 1253 GC patients were shown in Table 1. Of these patients, about half of the patients were older than 60, and the female‐to‐male ratio was 3:7. Gastric cancer most commonly occurred in the antrum or pylorus of the stomach (60%), then came cardia (21%), and body (19%) of the stomach. Most patients possessed tumors smaller than 5 cm (64%). The median tumor size was 4.1 cm (range 0.3–18 cm). With regard to the differentiation, most patients had tumors with low differentiation (77%), followed by median (21%) and high differentiation (2%). The lymphovascular and perineural invasion could be seen in approximately half of the patients. As for TNM stage of T stage, 56% of patients were classified as pT4 stage, whereas 28%, 8%, and 8% of patients were classified as pT1, pT2, and pT3 stage, respectively. As regards N stage, 37% of patients were classified as pN0 stage, whereas 26%, 19%, and 18% of patients were classified as pN3, pN2, and pN1 stage, respectively. R0 resections were performed in 98% of patients, whereas R1 or R2 resections were in 2% of patients. By the end of follow‐up, 30% patients died from GC or other reasons. In addition, we identified 97 patients who had TDs in the omentum of lesser curvature, 51 patients having TDs in the proximal omentum of greater curvature, and 15 patients with those in the distal omentum of greater curvature, respectively.

**TABLE 1:** Clinical characteristics of gastric cancer patients (*n* = 1253)

Variables	No. (%)
Gender	
Male	872 (70%)
Female	381 (30%)
Age (years)	
<60	636 (51%)
≥60	617 (49%)
Location	
Cardia, EGJ	266 (21%)
Body	326 (19%)
Antrum, pylorus	661 (60%)
Tumor size (cm)	
<5	802 (64%)
≥5	451 (36%)
Differentiation	
High	24 (2%)
Median	259 (21%)
Low	970 (77%)
Lymphovascular invasion	
Positive	567 (45%)
Negative	686 (55%)
Perineural invasion	
Positive	580 (46%)
Negative	673 (54%)
pT stage	
T1	354 (28%)
T2	94 (8%)
T3	98 (8%)
T4	707 (56%)
pN stage	
N0	466 (37%)
N1	226 (18%)
N2	241 (19%)
N3	320 (26%)
pTNM stage	
Stage I	332 (27%)
Stage II	292 (23%)
Stage III	629 (50%)
Stage IV	0 (0%)
TD in the omentum of greater curvature (distal)	
Positive	15 (1%)
Negative	1238 (99%)
TD in the omentum of greater curvature (proximal)	
Positive	51 (4%)
Negative	1202 (96%)
TD in the omentum of lesser curvature	
Positive	97 (8%)
Negative	1156 (92%)
Surgical margin	
R0	1224 (98%)
R1/R2	29 (2%)
Death	
Yes	380 (30%)
No	873 (70%)

Abbreviations: EGJ, esophagogastric junction; TD, tumor deposit.

### 3.2 Univariate and multivariate analyses of GC patients

Independent prognostic factors of GC patients were determined by univariate and multivariate analyses. We observed that age (hazard ratio [HR]: 1.616, *p* = 0.000), location (Cardia, esophagogastric junction vs. Antrum, pylorus, HR: 0.609, *p* = 0.000), tumor size (HR: 2.624, *p* = 0.000), differentiation (high vs. low, HR: 4.541, *p* = 0.033), lymphovascular invasion (HR: 2.782, *p* = 0.000), perineural invasion (HR: 2.702, *p* = 0.000), pT stage (T1 vs. T2, HR: 3.085, *p* = 0.000; T1 vs. T3, HR: 4.259, *p* = 0.000; T1 vs. T4, HR: 6.768, *p* = 0.000), pN stage (N0 vs. N1, HR: 1.645, *p* = 0.010; N0 vs. N2, HR: 2.662, *p* = 0.000; N0 vs. N3, HR: 6.768, *p* = 0.000), TDs in the distal omentum of greater curvature (HR: 3.507, *p* = 0.000), TDs in the proximal omentum of greater curvature (HR: 4.064, *p* = 0.000), TDs in the omentum of lesser curvature (HR: 2.057, *p* = 0.000), and surgical margin (HR: 2.057, *p* = 0.006) were significantly associated with OS in GC patients (Table 2). Multivariate cox regression analyses confirmed that age (HR: 0.685, *p* = 0.000), tumor size (HR: 0.767, *p* = 0.019), pT stage (T1 vs. T2, HR: 2.141, *p* = 0.017; T1 vs. T3, HR: 2.216, *p* = 0.007; T1 vs. T4, HR: 2.893, *p* = 0.000), pN stage (N0 vs. N3, HR: 3.233, *p* = 0.000), TDs in the proximal omentum of greater curvature (HR: 0.447, *p* = 0.000), and surgical margin (HR: 0.405, *p* = 0.001) were independent prognostic factors for OS (Table 2).

**TABLE 2:** Univariate and multivariate analyses of clinicopathological features and overall survival in gastric cancer patients

Variable	Univariate analysis			Multivariate analysis		
	HR	95% CI	*p*	HR	95% CI	*p*
Gender						
Male vs. Female	0.971	0.779–1.210	0.793			
Age (years)						
<60 vs. ≥60	1.616	1.317–1.982	**0.000**	0.685	0.554–0.847	**0.000**
Location						
Cardia, EGJ vs. body	1.127	0.865–1.469	0.375	1.176	0.891–1.551	0.253
Cardia, EGJ vs. antrum, pylorus	0.609	0.472–0.785	**0.000**	0.862	0.656–1.132	0.286
Tumor size (cm)						
<5 vs. ≥5	2.624	2.143–3.212	**0.000**	0.767	0.615–0.957	**0.019**
Differentiation						
High vs. median	3.237	0.792–13.221	0.102	1.236	0.296–5.155	0.772
High vs. low	4.541	1.131–18.236	**0.033**	1.113	0.269–4.614	0.882
Lymphovascular invasion						
Positive vs. negative	2.782	2.251–3.439	**0.000**	0.983	0.755–1.279	0.896
Perineural invasion						
Positive vs. negative	2.702	2.184–3.344	**0.000**	0.853	0.667–1.092	0.207
pT stage						
T1 vs. T2	3.085	1.731–5.501	**0.000**	2.141	1.148–3.992	**0.017**
T1 vs. T3	4.259	2.498–7.262	**0.000**	2.216	1.241–3.958	**0.007**
T1 vs. T4	7.180	4.843–10.645	**0.000**	2.893	1.795–4.662	**0.000**
pN stage						
N0 vs. N1	1.645	1.126–2.403	**0.010**	1.008	0.671–1.515	0.968
N0 vs. N2	2.662	1.901–3.727	**0.000**	1.422	0.966–2.091	0.074
N0 vs. N3	6.768	5.081–9.014	**0.000**	3.233	2.226–4.698	**0.000**
TD in the omentum of greater curvature (distal)						
Positive vs. Negative	3.507	1.923–6.395	**0.000**	0.774	0.412–1.453	0.425
TD in the omentum of greater curvature (proximal)						
Positive vs. Negative	4.064	2.891–5.711	**0.000**	0.447	0.308–0.650	**0.000**
TD in the omentum of lesser curvature						
Positive vs. Negative	2.057	1.227–3.448	**0.000**	0.821	0.602–1.120	0.214
Surgical margin						
R0 vs. R1/R2	2.057	1.227–3.448	**0.006**	0.405	0.236–0.695	**0.001**

*Note*: Cox regression analyses were done for all the above data.

Bold indicates that *p* values of these variables are <0.05.

Abbreviations: CI, confidence interval; EGJ, esophagogastric junction; TD, tumor deposit.

### 3.3 Correlation between TDs and clinicopathological features in GC patients

To evaluate the clinical importance of TDs in GC, patients were divided into TDs in the distal omentum of greater curvature, TDs in the proximal omentum of greater curvature, and TDs in the omentum of lesser curvature groups. Tables 3, 4, 5 summarize the relationship between clinicopathological features and selected variables. The presence of TDs in the distal omentum of greater curvature was correlated with higher possibility to have lymphovascular (*p* = 0.0357) and perineural (*p* = 0.0392) invasion, more advanced pT stage (*p* = 0.0084), higher possibility to have TDs in the proximal omentum of greater curvature (*p* = 0.0024), and TDs in the omentum of lesser curvature (*p* = 0.0040) and unfavorable survival (*p* = 0.0007) (Table 3).

**TABLE 3:** The correlation between TD in the omentum of greater curvature (distal) and clinicopathological features in gastric cancer

Variables	TD in the omentum of greater curvature (distal)		*p* ^a^
	Positive	Negative	
Gender			
Male	9	863	
Female	6	375	0.4081
Age (years)			
<60	10	626	
≥60	5	612	0.2997
Location			
Cardia, EGJ	3	263	
Body	6	320	
Antrum, pylorus	6	655	0.4449
Tumor size (cm)			
<5	6	796	
≥5	9	442	0.0606
Differentiation			
High	14	956	
Median	1	258	
Low	0	24	0.3282
Lymphovascular invasion			
Positive	11	556	
Negative	4	682	**0.0357**
Perineural invasion			
Positive	11	569	
Negative	4	669	**0.0392**
pT stage			
T1	0	354	
T2	0	94	
T3	0	98	
T4	15	692	**0.0084**
pN stage			
N0	2	464	
N1	3	223	
N2	2	239	
N3	8	312	0.0651
TD in the omentum of greater curvature (proximal)			
Positive	4	47	
Negative	11	1191	**0.0024**
TD in the omentum of lesser curvature			
Positive	5	92	
Negative	10	1146	**0.0040**
Surgical margin			
Positive	1	28	
Negative	14	1210	0.2976
Death			
Yes	11	369	
No	4	869	**0.0007**

*Note*: Bold indicates that *p* values of these variables are <0.05.

Abbreviations: EGJ, esophagogastric junction; TD, tumor deposit.

^a^ Pearson chi‐squared test or Fisher's exact test for all the other analysis.

**TABLE 4:** The correlation between TD in the omentum of greater curvature (proximal) and clinicopathological features in gastric cancer

Variables	TD in the omentum of greater curvature (proximal)		*p* ^a^
	Positive	Negative	
Gender			
Male	32	840	
Female	19	362	0.2777
Age (years)			
<60	19	617	
≥60	32	585	**0.0489**
Location			
Cardia, EGJ	6	260	
Body	15	311	
Antrum, pylorus	30	631	0.2405
Tumor size (cm)			
<5	24	778	
≥5	27	424	**0.0100**
Differentiation			
High	43	927	
Median	8	251	
Low	0	24	0.3708
Lymphovascular invasion			
Positive	38	529	
Negative	13	673	**<0.0001**
Perineural invasion			
Positive	39	541	
Negative	12	661	**<0.0001**
pT stage			
T1	1	353	
T2	1	93	
T3	1	97	
T4	48	659	**<0.0001**
pN stage			
N0	3	463	
N1	4	222	
N2	17	224	
N3	27	293	**<0.0001**
TD in the omentum of greater curvature (distal)			
Positive	4	11	
Negative	47	1191	**<0.0001**
TD in the omentum of lesser curvature			
Positive	18	79	
Negative	33	1123	**<0.0001**
Surgical margin			
Positive	0	29	
Negative	51	1173	0.2617
Death			
Yes	37	343	
No	14	859	**<0.0001**

*Note*: Bold indicates that *p* values of these variables are <0.05.

Abbreviations: EGJ, esophagogastric junction; TD, tumor deposit.

^a^ Pearson chi‐squared test or Fisher's exact test for all the other analyses.

**TABLE 5:** The correlation between TD in the omentum of lesser curvature and clinicopathological features in gastric cancer

Variables	TD in the omentum of lesser curvature		*p* ^a^
	Positive	Negative	
Gender			
Male	68	804	
Female	29	352	>0.9999
Age (years)			
<60	39	597	
≥60	58	559	**0.0343**
Location			
Cardia, EGJ	22	244	
Body	37	289	
Antrum, pylorus	38	623	**0.0077**
Tumor size (cm)			
<5	61	741	
≥5	36	415	0.8261
Differentiation			
High	80	890	
Median	17	242	
Low	0	24	0.2388
Lymphovascular invasion			
Positive	68	499	
Negative	29	657	**<0.0001**
Perineural invasion			
Positive	66	514	
Negative	31	642	**<0.0001**
pT stage			
T1	2	352	
T2	2	92	
T3	7	91	
T4	86	621	**<0.0001**
pN stage			
N0	10	456	
N1	17	209	
N2	27	214	
N3	43	277	**<0.0001**
TD in the omentum of greater curvature (distal)			
Positive	5	10	
Negative	92	1146	**0.0040**
TD in the omentum of greater curvature (proximal)			
Positive	18	33	
Negative	79	1123	**<0.0001**
Surgical margin			
Positive	4	25	
Negative	93	1131	0.2753
Death			
Yes	52	328	
No	45	828	**<0.0001**

*Note*: Bold indicates that *p* values of these variables are <0.05.

Abbreviations: EGJ, esophagogastric junction; TD, tumor deposit.

^a^ Pearson chi‐squared test or Fisher's exact test for all the other analyses.

With regard to TDs in the proximal omentum of greater curvature, it was positively related to older people (*p* = 0.0489), larger tumors (*p* = 0.0100), higher possibility to have lymphovascular (*p* < 0.0001) and perineural (*p* < 0.0001) invasion, more advanced pT stage (*p* < 0.0001) and pN stage (*p* < 0.0001), higher possibility to have TDs in the distal omentum of greater curvature (*p* < 0.0001), and TDs in the omentum of lesser curvature (*p* < 0.0001) and unfavorable survival (*p* < 0.0001) (Table 4).

As for TDs in the omentum of lesser curvature, it was positively concerned with older people (*p* = 0.0343), higher possibility to locate in upper sites like cadia and body(*p*=0.0077), higher possibility to have lymphovascular (*p* < 0.0001) and perineural (*p* < 0.0001) invasion, more advanced pT stage (*p* < 0.0001) and pN stage (*p* < 0.0001), higher possibility to have TDs in the distal omentum of greater curvature (*p* =0.0040), and TDs in the omentum of lesser curvature (*p* < 0.0001) and unfavorable survival (*p* < 0.0001) (Table 5).

### 3.4 Prognostic significance of TDs in GC patients

**FIGURE 2:**
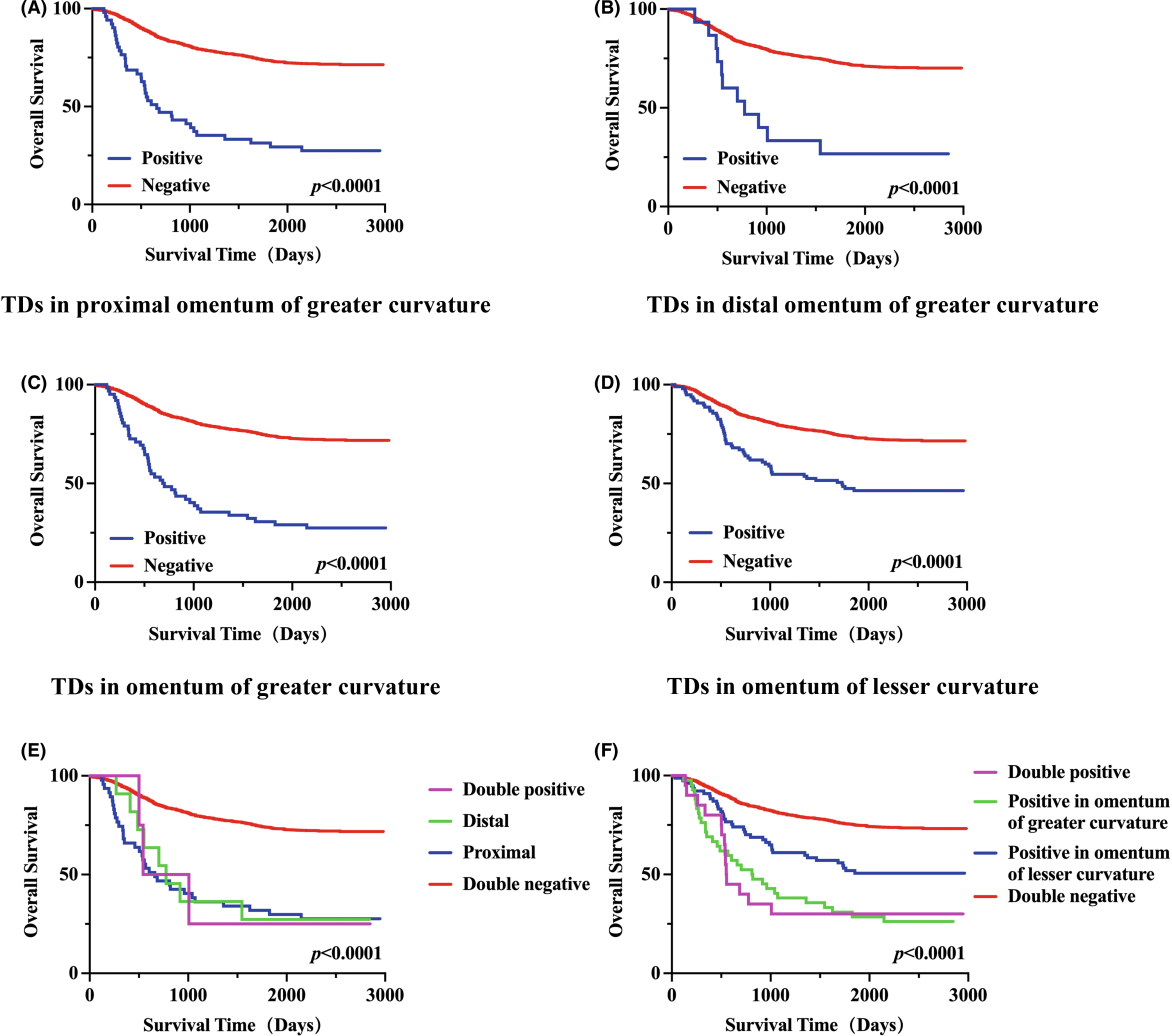
Kaplan–Meier curves of OS based on TDs of different positions in GC patients. (A) TD positivity in the proximal omentum of greater curvature (blue) versus no TDs (red). (B) TD positivity in the distal omentum of greater curvature (blue) versus no TDs (red). (C) TD positivity in the omentum of greater curvature (blue) versus no TDs (red). (D) TD positivity in the omentum of lesser curvature (blue) versus no TDs (red). (E) TD positivity in the proximal omentum of greater curvature (blue) versus TD positivity in the distal omentum of greater curvature (green) versus double positive (purple) versus double negative (red). (F) TD positivity in the omentum of greater curvature (green) versus TD positivity in the omentum of lesser curvature (blue) versus double positive (purple) versus double negative (red). GC, gastric cancer; OS, overall survival; TD, tumor deposit.

Kaplan–Meier curves were used to evaluate the effect of TDs on the prognosis of GC patients. We observed that patients with TDs in the proximal omentum of greater curvature (median OS, 1173 days vs. 2079 days, *p* < 0.0001) (Figure 2A) and TDs in the distal omentum of greater curvature (median OS, 1254 days vs. 2052 days, *p* < 0.0001) **(**Figure 2B) had shorter OS than those without them. In addition, as we combined these TDs into TDs in the omentum of greater curvature, we found that patients with TDs in the omentum of greater curvature (median OS, 1189 days vs. 2086 days, *p* < 0.0001) (Figure 2C) and TDs in the omentum of lesser curvature (median OS, 1620 days vs. 2077 days, *p* < 0.0001) (Figure 2D) both had shorter OS compared with other patients.

Subgroup analyses were adopted to further elucidate the prognostic role of TDs. It was found that patients with TDs in the omentum of greater curvature had similar OS, regardless of the positions of TDs (proximal or distal or both) (Figure 2E). Moreover, patients with TDs in the omentum of greater curvature had the worst prognosis, followed by patients with TDs in the omentum of lesser curvature and patients with no TDs (Figure 2F). It was worth noting that patients with TDs in the omentum of greater curvature had similar OS to those with TDs in the omentum of greater curvature and lesser curvature (Figure 2F).

### 3.5 Compositions of TDs in different TNM stages

**FIGURE 3:**
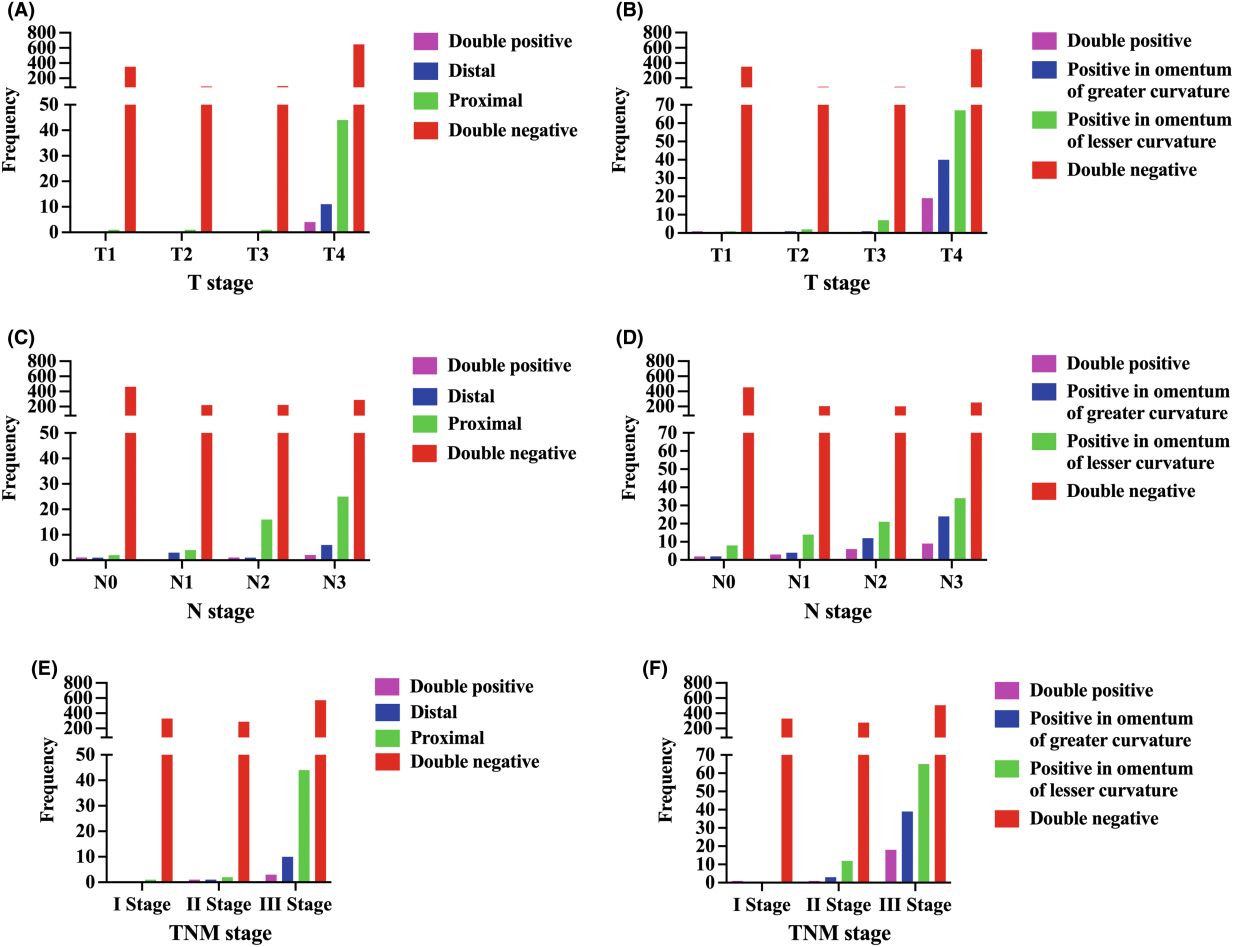
Compositions of TDs in different TNM stages. (A) Comparison of frequencies (y‐axis) of patients with TDs in the proximal omentum of greater curvature, patients with TDs in the distal omentum of greater curvature, patients with double positive TDs, and patients with no TDs in the omentum of greater curvature in relation to different T stages (x‐axis). (B) Comparison of frequencies (y‐axis) of patients with TDs in the omentum of greater curvature, patients with TDs in the omentum of lesser curvature, patients with double positive TDs, and patients with no TDs in relation to different T stages (x‐axis). (C) Comparison of frequencies (y‐axis) of patients with TDs in the proximal omentum of greater curvature, patients with TDs in the distal omentum of greater curvature, patients with double positive TDs, and patients with no TDs in the omentum of greater curvature in relation to different N stages (x‐axis). (D) Comparison of frequencies (y‐axis) of patients with TDs in the omentum of greater curvature, patients with TDs in the omentum of lesser curvature, patients with double positive TDs, and patients with no TDs in relation to different N stages (x‐axis). (E) Comparison of frequencies (y‐axis) of patients with TDs in the proximal omentum of greater curvature, patients with TDs in the distal omentum of greater curvature, patients with double positive TDs, and patients with no TDs in the omentum of greater curvature in relation to different TNM stages (x‐axis). (F) Comparison of frequencies (y‐axis) of patients with TDs in the omentum of greater curvature, patients with TDs in the omentum of lesser curvature, patients with double positive TDs, and patients with no TDs in relation to different TNM stages (x‐axis). TD, tumor deposit; TNM, tumor node metastasis.

The compositions of TDs in different TNM stages were analyzed to have a better understanding of TDs (Figure 3; Figure S1). It was observed that the positive rates of TDs were directly proportional to TNM stages. The higher the TNM stages were, the greater the possibility of TDs existing. TDs in the omentum of lesser curvature accounted for the largest proportion, followed by TDs in the proximal omentum of greater curvature and TDs in the distal omentum of greater curvature. Interestingly, we found that only patients classified as pT4 had TDs in the distal omentum of greater curvature (Figure 3A; Figure S1A), whereas no TDs were detected pathologically in those of T1–T3 patients.

## 4 DISCUSSION

Gastric cancer is an aggressive disease that remains one of the leading causes of cancer‐related death worldwide.^2^ Gastrectomy with D2 lymphadenectomy has been regarded as the standard surgical procedure for GC patients,^32^ with improved 5‐year OS rates of 23%–36% if perioperative chemotherapy is administered.^33^ Peritoneal metastasis represents the most common route of tumor dissemination in GC patients, especially when the tumor has infiltrated as far as the gastric serosa.^34^ Metastasis is most possibly caused by the presence of free cancer cells in the abdominal cavity.^35^ The omentum is a good place to harbor free cancer cells^25^ and is found to be a focus of macroscopic TDs in surgery. Based on this reason, routinely omentectomy is thought to be a kind of radical surgery for tumors with a high incidence of peritoneal seedings, such as GC and ovarian cancers.^36^ In the present work, to have a better understanding of the clinical significance of omentectomy, we retrospectively analyzed the incidence and clinical significance of TDs in different anatomical subregions of perigastric omentum of 1253 GC patients who underwent gastrectomy with total omentectomy.

Tumor deposits are found during routine pathologic examinations of about 10%–28% of resected GC specimens.^37^ In this study, TD positivity was 11.2%, which was quite consistent with the above literature. The differences in TDs positivity reported by literature may be due to racial and geographical changes. TD positivity is higher in tumors with larger sizes, deeper invasion, extended lymph node metastasis, and higher stage.^38^ Several studies have identified TDs as a significant prognostic factor indicating a particularly aggressive biological behavior. For bladder and cervical cancer, TDs correlate with a higher rate of local failure and shorter disease‐free survival (DFS) and OS.^39^ For breast cancer, TDs are proved to be an adverse prognostic factor of DFS.^40^ For GC, Sun et al.^41^ report that TDs are prevalent in tumors with a larger size, Borrmann type 4, lymphovascular infiltration, deeper infiltration, and a wide range of lymph node metastasis, and TDs are significantly correlated with patients' OS. In our study, we found that TDs in the omentum of greater and lesser curvature were associated with lymphovascular invasion, perineural invasion, advanced TNM stages, and unfavorable survival, which was consistent with the above literature and indicated the aggressive biological behaviors. In addition, TDs in the proximal omentum of greater curvature and in the omentum of lesser curvature correlated with older patients and larger tumors. Moreover, Kaplan–Meier curves demonstrated that patients with TDs in anywhere of omentum had shorter OS than those without them. Interestingly, as for the specific position, patients with TDs in the omentum of greater curvature had the worst prognosis, followed by patients with TDs in the omentum of lesser curvature and patients with no TDs. Multivariate Cox regression analyses identified TDs in the proximal omentum of greater curvature as an independent prognostic factor for OS. Currently, TDs are intaken into the TNM classification of colorectal cancers.^42^ Several studies have tried to incorporate TDs into the TNM classification of GC. Anup et al.^2^ find a significant difference in OS between pT1, pT2, pT3, and TDs and similarity between pT4 and TDs, suggesting that the prognosis of patients with TDs is almost the same as that of pT4 patients, regardless of the pT stage. Thus, they suggest that TDs should be included in the pT stages. Besides, when staging, patients with TDs should be treated differently from those without TDs. Lee et al.^43^ find that the presence of perigastric TDs is significantly associated with poor prognosis in the pN1, pN2, and pN3a subgroups and suggest that perigastric TDs be included in lymph node stage. They adopt the definition of lymph node metastasis in the colorectal cancer of the AJCC staging system and restage GC after excluding TDs from the count of lymph node metastasis. In Lee's study, there are 2 cases of pT1 cancer with TDs and 4 cases of pT2 cancer with TDs, representing only 0.9% (6 out of 653 cases). Therefore, TDs are clinically considered to have little impact on pT staging. Other researchers report that TDs are associated with hepatic and peritoneal metastases in patients with GC that TDs maybe indicated in the pM category.^37^, ^44^ Therefore, whether to incorporate TDs in the pT or pN or pM stages is still a matter of further study in GC.

The great omentum covers most of the abdomen viscera and is generally considered a barrier to protect the abdominal organs from outside attacks. Recent studies report that the omental adipocytes can promote cell proliferation and invasion in GC^45^ and ovarian cancers.^46^, ^47^ It is thought that omentectomy could prevent peritoneal recurrence by removal of so‐called “milky spots”.^48^, ^49^ However, it remains controversial whether GC patients can benefit from omentectomy. Several studies have demonstrated no differences in OS or DFS between complete and partial omentectomy in GC.^18^, ^26^, ^27^, ^50^, ^51^ On the contrary, complete omentectomy is associated with longer operating time, greater blood loss, and a higher risk of postoperative complications such as abdominal abscesses, ascites, anastomotic leakage, ileus, wound infections, and colonic and mesocolonic injuries.^26^, ^27^, ^52^ Patients undergoing partial omentectomy have a significantly shorter operation time and a higher serum albumin concentration on the first postoperative day,^18^ whereas a higher concentration of serum albumin on the first postoperative day is a better predictor of surgical outcome than other preoperative risk factors.^53^ Theoretically, residual great omentum can adhere to the inflammatory bowel and fill up part of anastomotic leakages and the macrophages residing in the great omentum may play an important role in removing residual tumors or free peritoneal tumor cells.^18^ According to the Japanese gastric cancer treatment guidelines, partial omentectomy (the omentum more than 3 cm away from the gastroepiploic artery) maybe performed in cases of pT1 and pT2, whereas total omentectomy is usually integrated into the standard gastrectomy for pT3 or deeper tumors.^54^ In our research, after studying the compositions of TDs in 1253 GC patients, we found that only patients classified as pT4 had TDs in the distal omentum of greater curvature, which indicated that partial omentectomy might be practicable for GC patients classified as T3 or shallower tumors.

In conclusion, we found that patients with TDs in the omentum of greater curvature had the worst prognosis, followed by patients with TDs in the omentum of lesser curvature and patients with no TDs. Besides, we found that only patients classified as pT4 had TDs in the distal omentum of greater curvature, which indicated that partial omentectomy might be practicable for GC patients classified as T3 or shallower tumors. This finding might contribute a lot to the modern medical decision‐making process.

## AUTHOR CONTRIBUTIONS

Yu Zhang put forward the problem. Qiaowei Lin and Yu Zhang designed the whole process. Qianming Bai diagnosed UPS in our patient cohorts as well as provided more detailed pathological information. Qiuyi Huang helped to improve the English writing of the manuscript. Yakai Huang and Jianpeng Gao collected the clinical information. Qiaowei Lin analyzed the data and wrote this article.

## FUNDING INFORMATION

This work was funded by the National Natural Science Foundation of China (81902392).

## CONFLICTS OF INTEREST

The authors declare that they have no potential conflict of interest.

## ETHICS APPROVAL STATEMENT

The study was approved by the ethics committee of the Shanghai Cancer Center, Fudan University and signed informed consent was obtained from each patient.

## Supporting information


Figure S1

## Data Availability

The data presented in this study are available on request from the corresponding author. The data are not publicly available due to privacy.
