# Supplementary figures and images for: Partial omentectomy maybe practicable for T3 or shallower gastric cancer patients
Source: Cancer Med. 2022 Jul 20;12(2):1204–16. doi: 10.1002/cam4.4980 (PMC9883580; doi:10.1002/cam4.4980)

**a**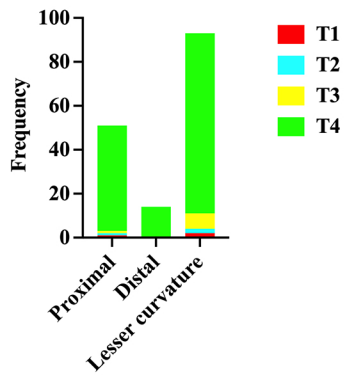**b**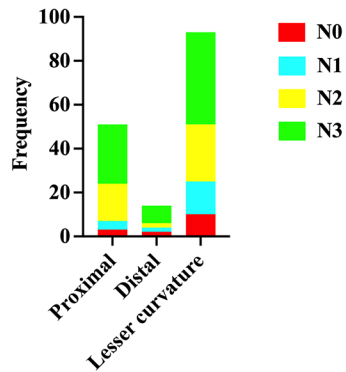**c**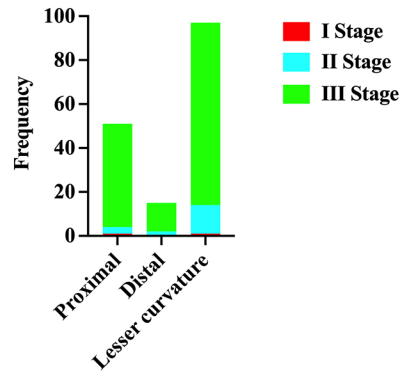

Supplement: Supplementary file 1 — Figure S1 [file CAM4-12-1204-s001.pdf]
